# Immigration as risk factor for non-suicidal self-injury and suicide attempts in adolescents in Germany

**DOI:** 10.1186/s13034-015-0065-4

**Published:** 2015-09-28

**Authors:** Paul L Plener, Lara M Munz, Marc Allroggen, Nestor D Kapusta, Jörg M Fegert, Rebecca C Groschwitz

**Affiliations:** Department of Child and Adolescent Psychiatry and Psychotherapy, University of Ulm, Steinhoevelstr. 5, 89075 Ulm, Germany; Department of Psychoanalysis and Psychotherapy, Medical University of Vienna, Vienna, Austria

**Keywords:** Non-suicidal self-injury, NSSI, Suicide attempts, Adolescents, Immigration, Migration

## Abstract

**Background:**

Whereas non-suicidal self-injury (NSSI) and suicide attempts (SA) are rather common among adolescents, the description of risk factors has often failed to take migration into perspective. Our study aimed to describe immigration status in adolescents with regards to their lifetime history of NSSI and SA.

**Methods:**

We carried out a population based study in a school community of ninth-graders (N = 452, mean age 14.85, SD 0.58) in southern Germany. Data were collected via adolescent self report on sociodemographic variables and on NSSI and SA using the Self Harm Behavior Questionnaire.

**Results:**

Adolescents born outside Germany showed an elevated rate of a lifetime history of NSSI and SA. When compared to German adolescents without a (family) history of migration (NSSI 19.16%, SA 3.24%), adolescents who were born in another country had an elevated risk for NSSI (42.86%, OR 3.36) and SA (17.86%, OR 6.78), which was higher than the risk of adolescents who had at least one parent who had emigrated from another country (NSSI 30.08%, OR 2.46 and SA 8.94%, OR 4.45).

**Conclusion:**

Our findings should inform intervention services and prevention programs for NSSI and suicidality in youth. Adopting such programs to include culturally sensible modules could improve the outcome in ethnically diverse adolescents.

## Background

Both non-suicidal self-injury (NSSI) and suicide attempts are rather common among German adolescents. Whereas worldwide lifetime prevalence rates of NSSI between 17 and 18% were reported from systematic reviews [1, 2], studies from Germany reported a lifetime prevalence rate of 25.6% in adolescents [3], and a 6-month prevalence rate of 14.6% [4]. Using criteria proposed in section 3 of the DSM-5 for NSSI disorder [5], a retrospective data analysis described a prevalence of 4% among German adolescents [6]. Recently, a large study comparing adolescent samples from 11 European countries (including Israel) reported a lifetime prevalence rate of 27.6% of “direct self-injurious behavior” (D-SIB: combining self-harming behaviors regardless of suicidal intent). Adolescents from Germany showed the second highest prevalence rate for occasional (22.9%) and for repetitive (12.3%) D-SIB [7].

With regards to suicidal ideation and suicide attempts, a large (n = 45,806) European study reported a median lifetime prevalence rate of 10.5% for suicide attempts in adolescents, with 30.8% reporting a history of self-harm thoughts [8]. In Germany, lifetime prevalence rates of suicidal ideation in adolescents were reported to be between 14.5% [9], 36.4% [3] and 39.4% [10], with a reported 6-month prevalence rate of 3.8% [11]. A lifetime history of suicide attempts was reported to be between 6.5% [3], 7.8% [9] and 9.0% [10].

Searching for predictors of NSSI, a recent systematic review of longitudinal studies described several factors, stemming from 32 longitudinal studies [12]. Among them were female gender, a history of previous NSSI, suicide attempts or suicidal ideation, and depressive symptoms. However, migration was not described being a risk factor for NSSI in these studies, due to the fact that it had not been included as a possible risk factor in most studies. However, migration in itself might be viewed as a combination of several stressors, for example the loss of cultural connectedness, the use of another language, the adaption to new norms and lifestyles, discrimination, peer alienation and changes in the socioeconomic status (for review [13]).

Literature about migration and suicidality in adolescents is still very scarce [13]. Furthermore, most studies attempting to further explore migration status as risk factor for NSSI and suicidal behavior have been conducted in the US. Borges et al. [14] reported from two nationally representative surveys about suicidal behavior being higher for Mexican immigrants who came to the US before the age of 12, as well as for US born Mexican Americans. Furthermore, the risk for suicide attempts nearly doubled (OR 1.97) for US born Mexican Americans. Interestingly, in a study on Boston youth, Borges et al. [15] described adolescents with a migration background not to be at higher risk for NSSI and suicidal ideation than US born youth. However, being discriminated due to one’s ancestry increased the risk of NSSI (OR 3.1) and suicidal ideation (OR 2.1) in US born youth with a background of migration. The authors of the study argued, that a dissonance between being born in a country and yet not being perceived as fully integrated could create a distress in these youths [15]. In addition, it was shown that US-born Latino adolescents were 2.87 times more likely to attempt suicide as Latino youth born in another country (i.e. first-generation youth). Third generation Latino youth (with US born parents) were even 3.57 times more likely to attempt suicide than first generation Latino adolescents [16]. Contrary to these findings, differences in rates of suicide attempts between different ethnic groups in a large (N = 15,180) US based Collaborative Psychiatric Epidemiological survey vanished to exist after adjusting for psychiatric disorders [17]. A Canadian study looking into suicides in youth between the age of 15 and 24, showed that immigrants´ suicide death rate was lower than the death rate of Canadian youth [18].

A European perspective has been reported based on data from the WHO/EURO Multicentre Study on Suicidal Behaviour, in which suicide attempt rates of adults were compared among 25 European centers. Overall, suicide attempt rates were higher in participants with a migration background when compared to the population of their host country. There was a strong correlation between suicide attempt rates among immigrants and suicide rates in their countries of origin (with the exception of Chileans, Turks, Ukrainians and Iranians) [19].

In a large Swedish study of 10,018 young adults between the ages of 18 and 29, non-European females with a migration background showed a higher rate of suicide attempts than their Swedish counterparts, which was especially pronounced in first generation non-European females (OR 3.52) in comparison to second-generation females with a migration background (OR 1.60) [20]. In another Swedish study of more than a million children, who were followed up prospectively, youth with both parents being born outside of Sweden showed higher rates of self-harm. However, these differences diminished after adjusting for socioeconomic status, but were still elevated in migrants from Finland, Western countries and children of mixed couples (one parent from Sweden, one from another country [21]). In a case–control study comparing 70 Turkish immigrants, who had attempted suicide and 70 Swiss suicide attempters, it could be shown, that the percentage of young (between the age of 15 and 25 years) Turkish women was higher than in the Swiss comparison group [22]. In addition it has been shown from a retrospective chart review of 210 children and adolescents (6–18 years of age) presenting after a suicide attempt to an Emergency Outpatient Clinic in Istanbul, that besides immigration to a foreign country, internal migration (migration from other parts of the same country with large cultural differences) also serves as a risk factor to choose a high risk method of suicide attempt [23]. High acculturation stress, along with immigration stress was also reported to be associated with a higher rate of self harming behaviors in a sample of 1,651 Hispanic adolescents [24].

Data about the association between NSSI, suicide attempts and migration background is scarcely available from adolescent samples in Germany. However, young adult women with a migration background have been shown to have elevated rates of suicide and suicide attempts [25]. A study on suicide attempts of adult women with Turkish origin in the Berlin region found high age-adjusted incidence rates of suicide attempts between 66.9 and 92.2/100,000, with highest rates in the age group from 18 to 24 [26]. One large, representative study of 44,610 adolescents showed immigration background to be a risk factor for suicide attempts, especially for adolescents from “Islamic imprinted countries” (being defined as “all countries whose culture is essentially influenced by Islamic theology” according to [10]) with an OR of 1.55 [10]. In a recent study following a cohort of 6,378 German repatriates from Russia for up to 20 years, it has been shown, that migration between the age of 11 and 20 increased the risk of committing suicide or dying from events of undetermined intent in males [27]. However, NSSI has not been assessed in these studies. Overall, migration can be viewed as under-researched risk factor for self-harming behaviors. Our aim was to specifically explore migration status as risk factor both for NSSI as well as suicide attempts. We adjusted for socioeconomic status, gender and age.

## Methods

The survey was conducted as part of a study focusing on motives and (especially social) risk factors for NSSI and suicidal behavior in adolescents [28]. Students were recruited from 9th grades of 10 schools (different types of schooling: vocational, intermediate and academic) by giving oral and written information in the classroom within a time period of 6 months. We chose ninth graders as in the German school system different types of schooling branch after ninth grade (some type of schooling ends after these grade, whereas others go on for 10 or 12 grades). Written information was provided for caregivers as both active parental written informed consent as well as active adolescents’ written assent was necessary for participation in the study. Participation in the study required a basic knowledge of German language, as questionnaires were only available in German. Of 748 eligible students in these classes, 656 were present at the day of assessment and 452 (68.9%) of the students and their caregivers consented to take part in the study. Due to German school regulations, no data were obtained from those not participating in the study. Of the 452 participating students (mean age 14.85, SD 0.58; age range 14–17), 209 (46.2%) were female. With regards to type of schooling, 15.9% visited a vocational school (“Hauptschule”), 45.6% a school with intermediate academic level (“Realschule”) and 38.5% a school with the highest academic level (“Gymnasium”).

Assessment was anonymous as it is expected that non-anonymous studies create a bias toward lower rates of NSSI [2] because of study participants fearing consequences of disclosure of suicidality or NSSI. Participants with NSSI or suicidal behavior could contact the study team using “contact cards” and ask them for help. Furthermore, information cards were administered listing contact details of regional mental health providers and counselling services. The study was approved by the Institutional Review Board of the University of Ulm and by the local school authorities.

### Measures

#### Demographic measure

The questionnaire assessed gender, age, occupation of parents and migration status. We assigned social class retrospectively by calculating a standard household income based on the students information about their parents occupation. Income approximations were based on data from the German federal statistics bureau. Migration status was assessed using three items asking for country of birth of students, their parents and the language spoken at home. Participants were defined as “born in another country” if their country of birth was not Germany. NSSI and suicide attempts were assessed using a German version of the Self Harm Behavior Questionnaire (SHBQ) [3, 29]. The SHBQ is a self-report measure with four subscales evaluating NSSI (‘Have you ever hurt yourself on purpose?’), suicidal ideation (‘Have you ever talked or thought about committing suicide?’), and attempted suicide (‘Have you ever attempted suicide?’), as well as suicide threats (‘Have you ever threatened to commit suicide?’). The instrument has been validated showing good internal consistency (Cronbach´s α between 0.89 and 0.96 for 4 subscales) and has been used in both American and German adolescent community samples [3, 30].

### Statistical analysis

For group comparisons, Chi square test was applied. Spearman correlation coefficients were calculated to examine associations between suicide attempts and NSSI with sociodemographic factors. Odds ratios for risk factors were based on logistic regression using SPSS version 21.0.

Results were only calculated for the subgroups “participant born in Germany” vs. “participant born in another country” as well as “both parents born in Germany” vs. “one or both parents born in another country”, as all other subgroups (i.e. divided by country of origin or gender of participants/parents) were too small for analyses. Results of those small sub-groups are descriptively presented in Table 1.

**Table 1:** Sociodemographic data of participants in relation to lifetime suicide attempts and NSSI

	Total	Suicide attempt	NSSI
Total	452 (100%)	18 (4.0%)	92 (20.4%)
Age [M (SD)]	14.85 (0.58)		
Gender (m/f)	243 (53.8%)/209 (46.2%)	7 (3.04%)/11 (5.56%)	31 (12.97%)/61 (29.76%)
Participants			
Born in Germany	424 (93.8%)	13 (3.24%)	80 (19.16%)
Born outside of Germany	28 (6.2%)	5 (17.86%)	12 (42.86%)
Born in Turkey	3 (0.7%)	2 (66.67%)	2 (66.67%)
Born in Russia (or former USSR)	5 (1.1%)	1 (20.00%)	3 (60.00%)
Born in another foreign country	20 (4.4%)	2 (10.00%)	7 (36.84%)
Both parents born in Germany	329 (72.79%)	7 (2.13%)	55 (16.72%)
At least one parent born outside of Germany	123 (27.21%)	11 (8.94%)	37 (30.08%)
Fathers			
Born in Germany	348 (77.0%)	13 (3.95%)	64 (18.71%)
Born outside of Germany	104 (33%)	5 (4.81%)	28 (26.92%)
Born in Turkey	35 (7.7%)	3 (9.38%)	12 (35.29%)
Born in Russia (or former USSR)	10 (2.2%)	1 (10.00%)	3 (30.00%)
Born in another foreign country	59 (13.1%)	1 (1.75%)	13 (22.41%)
Mothers			
Born in Germany	351 (77.7%)	9 (2.71%)	64 (18.55%)
Born outside of Germany	101 (32.3%)	9 (8.91%)	28 (27.72%)
Born in Turkey	33 (7.3%)	3 (10.00%)	10 (31.52%)
Born in Russia (or former USSR)	10 (2.2%)	1 (10.00%)	3 (20.00%)
Born in another foreign country	58 (12.8%)	5 (8.77%)	15 (26.31%)

## Results

Out of all participants, 28 (6.2%) were not born in Germany. Five adolescents were born in Russia and former Soviet Union countries and three in Turkey. Of the students´ parents, 104 fathers (33%) and 101 mothers (32.3%) were born in another country. In detail, 10 mothers and 10 fathers were born in Russia and former Soviet Union countries and 33 mothers and 35 fathers in Turkey (see Table 1).

In total, 92 (20.4%) of the adolescents reported a lifetime history of NSSI, and 18 (4%) of a suicide attempt. Whereas girls reported NSSI more frequently (f: 61, m: 31; Chi^2^ = 18,926 df = 1, p < 0.001) no statistical significant gender difference was found for a history of suicide attempts (f: 11, m: 7; Chi^2^ = 1,667 df = 1, p = 0.197). Methods of suicide attempts included: ingestion of less than 10 tablets (1), ingestion of more than 10 tablets (2), ingestion of two or more substances (1), direct bodily harm (8), asphyxiation, hanging or use of a weapon (3). Most of the adolescents with a history of a suicide attempt reported one attempt (46.67%), with less reporting two (33.22%), three (6.67%) or more than three (13.33%) attempts. The majority of attempts have been carried out within 1 year prior to the study (57.14%), 7.14% between 1 and 2 years before the study and 35.71% more than 2 years ago.

**Fig. 1:**
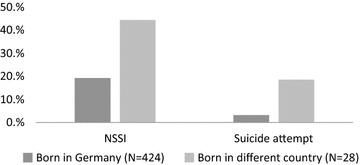
Lifetime prevalence of NSSI and suicide attempts in adolescents being born in Germany in comparison to those being born outside of Germany.

Adolescents, who were born outside of Germany reported a higher life time prevalence of NSSI and suicide attempts (see Fig. 1). These differences were statistically significant for NSSI (Chi^2^ = 9.796 df = 1, p = .002) and for suicide attempts (Chi^2^ = 14.654 df = 1, p < .001). Adolescents born outside of Germany only reported ingestion of more than 10 tablets (2) and direct bodily harm (3) as suicide attempt methods.

NSSI was initiated by 44 adolescents (47.83%) within the last year, by 40 (46.74%) 2 to 3 years ago, five (5.43%) had started 4 to 5 years ago and 2 (1.09%) 6 years or longer ago. For most of the adolescents reporting a history of NSSI the latest incident of NSSI had occurred within the last year prior to the study (60, 65.2%; 12.03% of adolescents born in Germany vs. 32.14% of the adolescents born outside of Germany). Of those adolescents reporting NSSI 42 (45.65%) reported a single incident, 19 (20.65%) had hurt themselves twice, 10 (10.87%) three times an 21 (22.83%) four times or more.

We found a positive association between suicide attempts and being born outside of Germany as well as a positive correlation between suicide attempts and having at least one parent who was not born in Germany (see Table 2).

**Table 2:** Correlation of lifetime history of suicide attempts and NSSI with socio-demographic data focusing on migration

	Suicide attempt		NSSI	
	r	p	r	p
Age	.08	.09	−.08	.08
Gender	−.06	.20	.21**	<.001
Family income	−.07	.18	.04	.47
Participant born in another country	.19**	<.001	.15**	<.002
One or both parents born in another country	.16**	<.001	.15**	<.002
Language at home other than German	.11	.03	−.11	.02
Language with friends other than German	−.03	.61	.01	.81

Bonferroni-corrected: significance level at p < .005.

Gender was not associated with migration background (r = .013, p = .788 for being born outside of Germany and r = −.061, p = .195 for at least one parent being born outside of Germany) and was therefore not included in the regression analyses as a possible predictor.

In a logistic regression analysis, both being born outside of Germany and having at least one parent who was born in another country explained 10% of the variance (see Table 3).

**Table 3:** Logistic regression analyses with suicide attempts and NSSI as dependent variables

	Coefficient	Standard error	Wald *p* value	Nagelkerkes R-Square
Dependent variable: suicide attempt				.100
Factor: one or both parents born in another country	1.14	.55	.038	
Dependent variable: NSSI				.045
Factor: one or both parents born in another country	.601	.268	.025	

Factors were drawn from correlation-analyses. Significance level at p < .05.

NSSI showed a positive association with being born outside of Germany, and having at least one parent who was born in another country. However, being born outside of Germany was not significant in the regression analysis and was therefore dropped from the model. Having at least one parent who was born in another country explained 4.5% of the variance. (see Table 3).

Compared to adolescents with no background of migration, those who were born outside of Germany had an elevated risk for NSSI (OR 3.36) and suicide attempts (OR 6.78). Those adolescents who had at least one parent who was born in another country also had higher risks for NSSI (OR 2.46) and suicide attempts (OR 4.45) compared to those whose parents were born in Germany.

## Discussion

We assessed a sample of 9th grade students from a community population in Germany with regards to NSSI and suicide attempts. The lifetime prevalence rate of 20.4% for NSSI seems comparable with former studies on NSSI in Germany [3] and is in the range of NSSI prevalence in adolescent community samples worldwide [1]. The rate of suicide attempts (4%) is lower than in other recent studies on German school populations (e.g. [9, 10] ) but closer to a previous study using the same assessment instrument [3], so that these differences might be explained by assessment methodology. Whereas a clear gender difference existed for NSSI, no statistically significant gender difference was found with regards to suicide attempts. This is contrary to former studies of suicide attempts in German minors [9] and is possibly attributable to low numbers of suicide attempts in our study. Family income was neither associated with NSSI, nor with suicide attempts. This may be due to the fact that economic burden is less of a risk factor for suicidality in the minors, than it is for adults, (e.g. in [31]), a finding that has been reported in other studies as well [32].

We aimed to evaluate whether migration should be understood as risk factor for NSSI or suicide attempts in adolescents. In summary, our findings support the notion that migration serves as a risk factor for both. We were able to show that both, being born outside of Germany as well as having one parent with migration background serves as risk factor for NSSI and suicide attempts. This shows that even in the second generation of migrants, these factors seem to play a relevant role in influencing the risk for self-harming behaviors. The findings of elevated rates of suicide attempts in adolescents with migration background is in line with recent work from another German sample [10].

Adolescents of whom at least one parent was born outside of Germany had elevated odds of a lifetime history of suicide attempts (OR 4.45), an effect, that was even more pronounced in adolescents being born outside of Germany (OR 6.78). This is in line with findings of elevated risk of suicide attempts in females with non-European migration background in a large population based study from Sweden [20]. However, these results were contradicting findings from the US, reporting higher rates of suicide attempts in second and third generation Latino youth [16]. These differences might be explained by the specific situation of Latino youth in the US, with the Swedish situation being more comparable to the situation in Germany. The finding of higher rates of NSSI and suicide attempts in adolescents with a first generation migration background could be due to possible traumatic events acquired throughout the experience of cultural and social disruption when moving to another country. However, due to the small numbers in our sample, differences between these groups have to be interpreted with extreme caution.

As stressors are influential for the development of NSSI (such as for example, in the integrated theoretical model of NSSI by Nock [33]), increasing stress can lead to higher levels of NSSI. With respect to suicidality, migration could contribute to a feeling of perceived burdensomeness (as in Germany some groups of immigrants are not allowed to work for a certain time span, therefore being reliant on social welfare) and thwarted belongingness (e.g. feeling isolated due to language or cultural barriers or cultural stress (see [23]). Both of these factors contribute to an increased risk for suicide, based on the interpersonal psychological theory of suicide by Joiner [34]. Referring to these theoretical models, it seems reasonable to expect a higher risk for both NSSI and suicidality in adolescents with a background of immigration.

Several limitations do apply when interpreting our findings. First, we used an anonymous self-report survey that was announced as a study of NSSI, suicidality and social influences. As was shown by Swanell et al. [2], both factors (anonymity and announcement as a study of NSSI) could influence the rates of NSSI (and possibly suicidality) towards a higher prevalence. However, as we wanted to ensure the possibility to answer truly without having to fear possible consequences, we chose the given procedure. Relying on self-report could create a recall bias between groups. Second, the SHBQ does not allow to further define suicide attempts by chronicity or severity, so that there might be the possibility that differences in suicidality between groups might have not been detected. Third, as both the consent and assent forms as well as the information sheets and the questionnaires were only available in German, this might have created a bias towards non-participation of adolescents with language barriers, or of adolescents whose parents were not able to understand the provided information. Fourth, due to school authority regulations we were not able to collect data from non-participants, therefore not being able to provide an analysis of adolescents, who (or their parents) chose not to participate in this study. Fifth, in our study, migration was only measured in terms of country of origin (both of the participants and their parents) and language spoken at home. We did not assess subjective feelings of discrimination due to migration status, which could have been useful to further analyze the ongoing psychological mechanisms in more detail. Sixth, variance explained by the main factors being born in another country, and at least one parent being born in another country, was quite low. This is probably due to the vast number of possible predictors of NSSI and suicide attempts which have been found in other studies (i.e. psychiatric symptoms, social support, adverse life-events, etc.) and have not been assessed in this study. Furthermore, the factors age at immigration, number of moves, move distance, duration of immigration status, degree of depression and somatization of adolescents and parents, actual or former medication or psychotherapeutic interventions or inpatient therapies were not assessed. These factors might have influenced the rates of NSSI and suicidality for some of the participants. Results of this study therefore only focus on a small aspect of possible predictors of NSSI and suicidality. However, due to high odds-ratios found in this study, results still seem to be relevant to research and prevention of NSSI and suicidality. Seventh, the sample of immigrants is not representative of the total German foreign born population.

To our knowledge, this is the first study analyzing migration background with regards to NSSI and suicide attempts in adolescents living in Germany. Our findings seem—at least in part—comparable to other studies in youth who experienced immigration in the first or second generation in Europe. It is unclear, whether these higher rates of suicide attempts will lead to a higher rate of committed suicides in later years, but evidence from a large sample of German repatriates from Russia suggests, that this could be the case as higher rates in males, who immigrated between the ages of 11 and 20 were reported [27]. The median lag time between migration and suicide was 8 years [27], so that there seems to be a need for prevention campaigns especially addressing adolescents or young adults with a history of immigration. Showing the scope of the problem, it seems necessary to include a culturally informed migration perspective in NSSI and suicidality prevention programs tailored for adolescents and young adults. With regards to intervention in suicidal crises, a program targeting Turkish women (with the use of telephone hotlines or training of lay persons) from Germany seemed promising [23]. However, so far these intervention procedures have only been applied in certain regions, mostly targeting adults. Our findings underscore the need for widening tailored approaches for adolescents with migration background in Germany.
